# Preoperative risk evaluation and optimization for patients with liver disease

**DOI:** 10.1093/gastro/goae071

**Published:** 2024-07-03

**Authors:** Sameer Bhalla, Brendan Mcquillen, Edward Cay, Nancy Reau

**Affiliations:** Internal Medicine, Rush University Medical Center, Chicago, IL, USA; Internal Medicine, Rush University Medical Center, Chicago, IL, USA; Internal Medicine, Rush University Medical Center, Chicago, IL, USA; Internal Medicine, Division of Digestive Diseases, Section of Hepatology, Rush University Medical Center, Chicago, IL, USA

**Keywords:** chronic liver disease, surgical complications, acute hepatitis

## Abstract

The prevalence of liver disease is rising and more patients with liver disease are considered for surgery each year. Liver disease poses many potential complications to surgery; therefore, assessing perioperative risk and optimizing a patient’s liver health is necessary to decrease perioperative risk. Multiple scoring tools exist to help quantify perioperative risk and can be used in combination to best educate patients prior to surgery. In this review, we go over the various scoring tools and provide a guide for clinicians to best assess and optimize perioperative risk based on the etiology of liver disease.

## Introduction

It is estimated that 1.5 billion people have chronic liver disease (CLD) worldwide [1]. CLD largely comprises metabolic dysfunction-associated steatotic liver disease (MASLD), hepatitis B, hepatitis C, and alcoholic liver disease. However, CLD can sometimes progress to cirrhosis and a study published in 2017 estimated that 112 million people worldwide have compensated cirrhosis [2]. Since the prevalence of CLD is increasing (due to the increase in incidence of MASLD and increase in life expectancy of patients with liver disease) [3], patients with liver disease are more likely to be considered for surgeries other than liver transplant and require careful preoperative consideration [4]. Although some liver diseases increase perioperative risk, most of the risk is concentrated in those patients with cirrhosis. Surgery can also lead to hepatic decompensation in a previously stable patient. In this review, we will discuss the risks of surgery in patients with liver disease, how to assess perioperative risk in patients with acute and CLD, limitations to current guidelines, and future considerations in assessing perioperative risk.

### Risks of surgery in patients with live disease

The pathophysiology of liver disease can result in increased risk for anesthesia, surgery, and postoperative healing, and therefore patients with cirrhosis have a three times increased risk of perioperative mortality compared with patients without cirrhosis [5–7]. This increased risk is due to a multitude of factors related to the pathophysiology of liver disease and cirrhosis. For example, patients with CLD may have difficulty in metabolizing commonly used anesthetics that have high hepatic clearance [8, 9]. Portal hypertension results in the formation of collateral circulation, thus increasing bleeding risk intraoperatively. Additionally, vasodilation from impaired arterial autoregulation in liver disease can cause hypotension intraoperatively and induce renal dysfunction. Patients with liver disease often have thrombocytopenia, splenic sequestration, and/or sarcopenia, resulting in a higher risk of infection, bleeding, clotting, and poor wound healing postoperatively [10–12]. Hepatic decompensation and worsening portal hypertension are also possible postoperative complications that must be considered. Due to these increased risks, it is important for patients with liver disease to undergo thorough preoperative evaluation by a multidisciplinary team of hepatologists, anesthesiologists, and surgeons to risk-stratify and optimize patients prior to surgery. The American Gastroenterological Association (AGA) has also released guidelines to best predict surgical outcomes and optimize patients with cirrhosis prior to surgery [13]. However, these guidelines do not apply to patients with acute liver disease or other forms of CLD and do not incorporate newer scoring tools used for assessing perioperative risk. Therefore, this article aims to provide clinicians with updated considerations and tools to assess perioperative risk in patients with liver disease.

**Figure 1.**
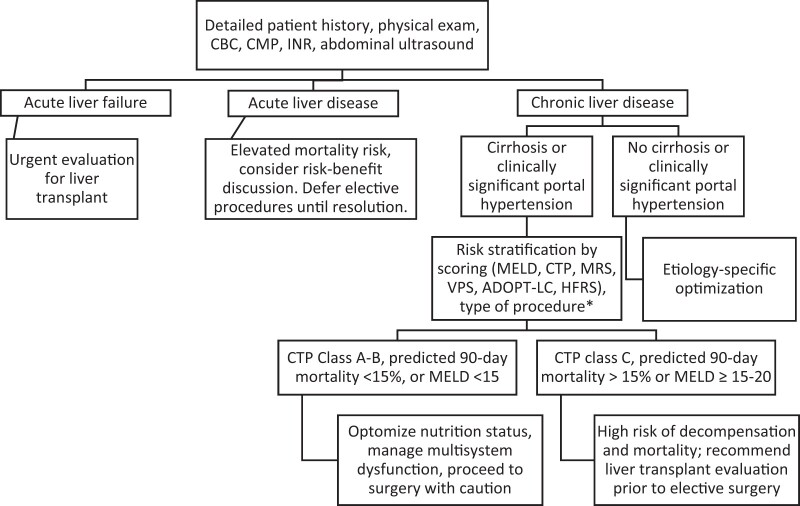
Approach to patients with liver disease undergoing surgery. ^a^High-risk procedures include open abdominal, cardiothoracic, major orthopedic, and abdominal wall surgery. CBC = complete blood count, CMP = comprehensive metabolic panel, INR = international normalized ratio, MELD = Model for End-Stage Liver Disease, CPS = Child–Pugh score, MRS = Mayo Risk Score, VPS = VOCAL-PENN score, ADOPT-LC = Adequate Operative Treatment for Liver Cirrhosis, HFRS = Hospital Frailty Risk Score.

The severity of liver disease is the strongest predictor of postoperative outcomes, but the type of surgery also significantly alters perioperative risk. At this time, there are multiple risk stratification tools that can help determine operative risk in patients with liver disease, each with its own strengths and weaknesses. Prior to use of these risk assessment tools, baseline assessment should always be completed to identify the possible presence of cirrhosis with a low threshold for further imaging or liver stiffness measurement, as patients with compensated advanced chronic diseases can have normal liver function tests [14]. Figure 1 depicts a flowsheet to evaluate patients with liver disease who are undergoing surgery.

### Baseline assessment

Baseline assessment should begin with a detailed history that focuses on risk factors for acute or CLD. This includes review of past medical history, family history, metabolic risk factors, current medications, social history (alcohol use, recreational drug use, tattoos), sexual history, history of blood transfusions, and travel history. Physical exam should focus on identifying features of portal hypertension and liver disease including jaundice, scleral icterus, spider angiomata, gynecomastia, splenomegaly, caput medusae, ascites, edema, or palmar/plantar erythema. Laboratory testing should begin with complete blood count, comprehensive metabolic panel, and international normalized ratio (INR).

If the above assessment is concerning for liver disease (i.e. low platelets, elevated liver enzymes, low albumin, elevated bilirubin, or elevated INR), further testing may be warranted prior to surgery. Abdominal ultrasound is useful to evaluate the size and texture of the liver, the presence of splenomegaly, and/or the presence of ascites. Depending on the results of the abdominal ultrasound, elastography may be useful to assess liver stiffness and aid in the diagnosis of cirrhosis. In some cases, transjugular pressure measurements may be pursued to assess the degree of portal hypertension. Identifying the presence of cirrhosis in patients with CLD is crucial, as it greatly increases perioperative risk. If there is uncertainty about the diagnosis of cirrhosis when using non-invasive testing, liver biopsy is the gold standard and would be a useful next step prior to surgery. Until formal evaluation and optimization of liver disease has been accomplished, elective surgeries should be delayed [15].

### Key takeaways

- Prior to surgery, clinicians should perform a detailed history and physical exam that aims to identify the presence of acute liver disease, CLD, and/or portal hypertension.
- Platelet count of <150,000 per microliter may reflect portal hypertension and requires additional assessment.

## Scoring tools

### Child–Turcotte–Pugh and model for end-stage liver disease-sodium

Commonly used scoring tools are summarized in Table 1. Traditional scoring tools include Child–Turcotte–Pugh (CTP) and Model for End-Stage Liver Disease-Sodium (MELD-Na), and have long been used for prognostication, but can overestimate surgical risk because they do not take into account the specific type of surgery or etiology of the liver disease [16]. Additionally, the MELD score has undergone revisions (MELD vs MELD-Na vs MELD 3.0) and prognostication studies over the years have used different variations of the scoring tool. CTP class is determined using albumin, bilirubin, INR, ascites, and encephalopathy whereas the MELD-Na score uses sodium, creatinine, bilirubin, and INR. Previous studies have shown perioperative mortality of 10% for CTP class A, 30% for class B, and 80% for class C [17]. In one retrospective study of 195 patients with cirrhosis undergoing surgeries, a MELD-Na score of >26 corresponded to a postoperative mortality of >90% [18]. CTP and MELD-Na were found to perform similarly [18]. The etiology of liver disease has also been found to predict postoperative mortality [19], which is why newer dedicated surgical risk prediction tools such as the Mayo Risk Score (MRS) were developed.

**Table 1.** Commonly used scoring tools

Scoring system	Components	Operative risk score	Validation
Child–Turcotte–Pugh (CTP)	Albumin, bilirubin, international normalized ratio, ascites, and encephalopathy	Mortality of 10% in class A, of 30% in class B, and of 80% in class C	Patients with cirrhosis
Model for End-Stage Liver Disease (MELD)—Sodium	Sodium, creatinine, bilirubin, and international normalized ratio	A 1% increase in mortality for each one-point increase in the MELD score from 5 to 20 and a 2% increase in mortality for each one-point increase in the MELD score above 20. MELD score <10, 10–14, and >14 may correspond to CTP class A, B, and C, respectively. MELD-Na score >26 corresponded to a postoperative mortality rate of >90%	Patients with cirrhosis
Mayo Risk Score	American Society of Anesthesiologists physical status classification, INR, total bilirubin, creatinine, age, and etiology of liver disease	https://www.mayoclinic.org/medical-professionals/transplant-medicine/calculators/post-operative-mortality-risk-in-patients-with-cirrhosis/itt-20434721	Patients with cirrhosis undergoing orthopedic, cardiovascular, or major abdominal surgery
VOCAL-PENN score	Type of surgery to be performed, age, BMI, bilirubin, platelet count, albumin, etiology of liver disease, fatty liver disease, and American Society of Anesthesiologists classification	https://www.vocalpennscore.com/	Predicts 90-day postoperative hepatic decompensation patients in the Veterans Health Administration with cirrhosis
Adequate Operative Treatment for Liver Cirrhosis Score	Age, Child–Turcotte–Pugh class, co-morbidities, and duration of anesthesia	https://apps.apple.com/uy/app/adopt-lc-score/id976986669 https://apkpure.com/adopt-lc-score/jp.cureapp.handlinglivercirrhosis	Japanese patients with cirrhosis undergoing major surgical procedure
Hospital Frailty Risk Score	International Statistical Classification of Diseases and Related Health Problems, Tenth Revision (ICD-10) diagnostic codes to group patients into low, intermediate, and high frailty risk	N/A	Hospitalized patients aged >75 years

### MRS

The MRS takes into account the American Society of Anesthesiologists (ASA) physical status classification, INR, total bilirubin, creatinine, age, and etiology of liver disease [20]. The study found that 1- and 3-month mortality increased by 14% with each one-point increase in the MELD score but, when externally validated, it was found to overestimate the 1-year mortality after surgery [21].

### VOCAL-PENN score

The VOCAL-PENN score (VPS) (www.vocalpennscore.com) further attempts to individualize perioperative risk by incorporating the type of surgery to be performed with the age, BMI, bilirubin, platelet count, albumin, etiology of liver disease, fatty liver disease, and ASA classification. This scoring system was originally developed from a large cohort of patients in the Veterans Health Administration (VA) and, when validated internally, showed superior discrimination compared with MELD-Na, CTP, and MRS at 30, 90, and 180 days [22]. It also was externally validated in two non-VA health systems and can predict 90-day postoperative hepatic decompensation [11].

### Adequate operative treatment for liver cirrhosis

Another tool, the Adequate Operative Treatment for Liver Cirrhosis (ADOPT-LC) score, was developed from a study including 2,197 patients in a Japanese nationwide database who underwent elective or emergency surgery to predict the in-hospital mortality of cirrhotic patients following major surgical procedures [23]. It uses patient age, CTP class, co-morbidities, and duration of anesthesia, and was found to outperform the CTP score in this cohort. Although the ADOPT-LC score was strongly predictive of inpatient mortality (sensitivity 90.5%, specificity 69.7%), it was not validated in multiple cohorts worldwide.

### Hospital Frailty Risk Score

Lastly, assessing patient frailty has also been shown to predict postoperative mortality, length of stay, and 90-day readmission. One study, also examining patients with cirrhosis in the VA, found that an increased Hospital Frailty Risk Score (HFRS) was a significant and independent predictor of postoperative mortality across multiple major surgery categories [24]. When incorporated with the MELD-Na or CTP, the HFRS improved perioperative risk prediction. Given that patients with cirrhosis are prone to frailty (sarcopenia, decompensations, comorbid conditions), HFRS can serve as a useful predictor for clinicians to tailor risk assessment to individuals and best educate patients.

### Key takeaways

- Multiple scoring tools exist to aid clinicians in assessing perioperative risk in patients with liver disease. Each scoring tool has its strengths and weaknesses, and can be used in conjunction to better gauge an individual patient’s perioperative risk.
- MELD and CTP are well-validated scoring tools and should be calculated for every patient with CLD who is considering surgical intervention.
- The VPS incorporates the type of surgery and has been shown to be superior to MELD and CTP in predicting risk.

When assessing perioperative risk in patients with liver disease, the etiology and acuity of the liver disease should be considered, as certain diseases confer additional risk. Here, we will discuss acute vs CLD and the specific risks and recommendations regarding surgery in each case.

## Acute liver diseases

Acute liver disease is considered a relative contraindication for surgery due to the risk of worsening with operative intervention and anesthesia, increased risk of bleeding, and increased risk of hepatic encephalopathy. Acute liver diseases include acute viral hepatitis, drug-induced hepatitis, alcohol-associated hepatitis (AH), autoimmune hepatitis, and acute liver failure (ALF). Of these, acute viral hepatitis, AH, and ALF have been identified as contraindications for elective surgery [25, 26]. For these reasons, it is recommended that any non-urgent operative intervention should be postponed until resolution or treatment [25, 27]. However, there is a notable lack of research in this area, and the evidence supporting current recommendations predates modern diagnostic practices, surgical techniques, and medical management. For example, one such study examined outcomes of diagnostic laparotomy in patients with suspected extrahepatic biliary obstruction who were subsequently found to have hepatitis of presumed drug or viral origin between the years 1950 and 1961 [28]. In another study, patients with alcoholic hepatitis who underwent open liver biopsy had increased mortality compared with those who underwent percutaneous needle aspiration between the years 1966 and 1970 [29]. Therefore, current recommendations are based on evidence from small cohorts of patients who underwent laparotomy and open liver biopsy many years ago [25, 28–31]. It is reasonable to postpone any elective surgery until the patient is optimized.

Scoring tools including the VPS, MRS, ADOPT-LC, HFRS, CTP, and MELD-Na have been validated for predicting postoperative mortality in patients with cirrhosis, but none has been validated in patients with acute liver disease. There are no known predictive models for postoperative mortality in non-cirrhotic patients with ongoing acute liver disease [22].

### ALF

In the USA, ALF is defined as liver injury of duration <26 weeks with associated coagulopathy (INR >1.5) and hepatic encephalopathy. Moreover, individuals must not have a history of pre-existing liver disease or cirrhosis. However, exceptions to this requirement are made for acute presentations of advanced fibrotic disease including Wilson’s disease, autoimmune hepatitis, and Budd–Chiari syndrome [32]. ALF progresses rapidly, is often characterized by multisystem dysfunction, and carries a high rate of morbidity and mortality [32–34]. For these reasons, ALF is a contraindication for any operative intervention except for emergent liver transplant [25].

### AH

Hospitalizations for alcohol-related liver disease are increasing in the USA [35]. AH is an acute presentation of alcohol-related liver disease that can be seen in patients without cirrhosis. It is characterized by rapid-onset jaundice and elevated aminotransferases, with asprtate aminotransferase (AST): alanine aminotransferase (ALT) >1.5 in the vast majority of patients [36]. The presence of AH is a contraindication to elective surgery. In addition to jaundice, associated symptoms may include malaise and tender hepatomegaly [37]. Other causes of jaundice and liver disease such as viral hepatitis, hepatocellular carcinoma, portal vein thrombosis, biliary obstruction, and drug-induced liver injury (DILI) should be ruled out. Liver biopsy provides definitive diagnosis and is recommended if the diagnosis remains uncertain [38]. This may be relevant when trying to differentiate biliary obstruction from hepatic dysfunction. However, liver biopsy is less effective for predicting mortality than non-invasive scoring systems such as MELD and Maddrey’s Discriminant Function (DF) [39]. Several prognostic scoring systems have been validated for use in AH. They can predict short-term mortality, guide decision-making for steroid therapy, and identify non-responders to steroid therapy. These models may help to guide decision-making for urgent operative interventions if they must be completed prior to resolution of AH but have not been validated specifically to gauge surgical risk. These scoring systems include MELD, CTP, the Glasgow Alcoholic Hepatitis Score (GAHS), Age-Bilirubin-INR-Creatinine (ABIC) score, and Lille Score. The presence or absence of cirrhosis and/or decompensation should guide the choice of scoring system. The DF, ABIC score, Lille Model, and GAHS were developed specifically for AH. ABIC and GAHS better predict mortality than DF [40, 41]. The ABIC score more accurately predicts 90-day mortality than MELD, DF, or GAHS, and is the only useful model for predicting 1-year mortality [41–43]. The Lille Model is a dynamic model that assesses response to steroid therapy. It is also highly predictive of 6-month survival [41, 44, 45]. Infection risk is an important consideration in the preoperative evaluation in patients with AH. Patients with AH may have multiple risk factors for infection including ongoing steroid treatment and underlying immune dysfunction due to alcoholic liver disease [46]. Therefore, non-life-threatening surgery should be deferred in AH if possible. If, however, surgery is deemed necessary, then the patient should also be assessed for liver transplant given the high risk of further decompensation.

### DILI

DILI is a diagnosis of exclusion made via thorough history and physical and laboratory evidence of liver injury. The R-value can assist in characterizing the pattern of liver injury at presentation in order to guide further evaluation and exclude other etiologies. Notably, DILI can present with hepatocellular, cholestatic, or mixed pattern. DILI typically occurs within 6 months of use of the offending drug but can occur later. Antibiotics and anti-epileptics are the most common causes, and DILI secondary to herbal and dietary supplements is becoming increasingly common [47–49]. Many offending agents have been characterized by latency, injury pattern, and histologic features. LiverTox, which is a free online database containing >1,200 agents known to cause DILI, can be invaluable in diagnosis. Liver biopsy is not required for diagnosis and not always definitive. However, biopsy may assist with distinguishing DILI from other etiologies such as autoimmune hepatitis [49].

DILI prognosis varies widely. Recovery within 6 months of withdrawal of the offending drug occurs in most cases, although 10% of patients progress to ALF. Chronic liver injury beyond 6 months can also occur [49]. African Americans are at greater risk of a range of adverse outcomes than Caucasian patients [50]. Pre-existing liver disease is associated with greater mortality [49]. Patients with cholestatic DILI are at greater risk of developing chronic liver injury, whereas patients with hepatocellular DILI and jaundice have greater risk of mortality and liver transplant. Interestingly, mixed injury has been associated with milder disease course [49]. Several predictive models for all-cause mortality in DILI patients exist and may influence clinical decision-making in patients under consideration for surgery. These include MELD, DrILTox ALF Score, Ghabril model, Hy’s Law, nHy’s Law, and Robles–Diaz Model. The Ghabril model is the newest, has been validated for prediction of 6-month mortality, and is recommended by American College of Gastroenterology clinical practice guidelines [41]. More recently, the above models were compared directly in two independent DILI cohorts in China. The Ghabril model was the most effective and was found to be an accurate predictor of mortality at 28 days, 90 days, 6 months, and 12 months [41].

If patient is found to have DILI prior to elective surgery, the offending agent should be stopped and the surgery should be deferred until DILI resolves. DILI usually resolves in a few weeks.

### Key takeaway

- Acute liver diseases including ALF, AH, and DILI are contraindications to surgery. In these instances, surgery should be deferred until resolution of these acute conditions.

## CLD

An estimated 1.5 billion persons have CLD worldwide and the incidence of CLD and cirrhosis has increased by 13% since 2000 [4, 51]. Despite this increased prevalence, patients are living longer due to early detection and improved medical management. This increased lifespan has also resulted in an increased number of patients with CLD being considered for non-transplant surgery and thus calculating perioperative risk is important in guiding patient safety.

### Cirrhotic liver disease

Compared with the general population, patients with cirrhosis are at an increased risk of perioperative complications and decompensation, including ascites, volume overload, encephalopathy, bleeding, thromboembolism, renal impairment, and death after any invasive surgical procedure, due to portal hypertension and impaired hepatic synthetic function [52]. Although patients with prior history of decompensation are at highest risk of decompensation after surgery, patients with well-compensated cirrhosis can still experience decompensation postoperatively [13]. All patients with cirrhosis should be risk-stratified prior to undergoing surgical procedures. This includes the anatomic location of the surgery, as intra-abdominal procedures, such as hepatobiliary surgery, as well as cardiothoracic surgery have an especially higher surgical risk. While there are no absolute values that exclude these patients from surgery, patients with Child–Pugh class C or MELD of >20 points have a high risk of decompensation and mortality [13].

### Key takeaway

- Consider preoperative liver transplant for elective surgery evaluation if the predicted 90-day mortality is >15% or MELD is ≥15–20.

## Type of surgery

In patients with CLD, the type of surgery impacts postoperative mortality. Evidence has shown that cirrhotic patients who underwent laparoscopic abdominal surgery had lower mortality than those who underwent open abdominal surgery. Additionally, mortality was highest for patients who underwent open abdominal surgery, followed by those undergoing cardiothoracic surgery, major orthopedic surgery, and abdominal wall surgery. Though surgery has traditionally deferred in patients with cirrhosis, it is important to consider elective surgery before emergent indications, as emergency surgery carries a much higher risk.

### Cholecystectomy

Gallstones are twice as common in cirrhotic patients as in the general population [53]. Previous studies suggested that patients with cirrhosis should not undergo elective cholecystectomy due to increased risk of mortality and conversion to open procedure [54]. This was supported by the fact that patients with cirrhosis who undergo cholecystectomy tend to have more postoperative complications compared with those without cirrhosis. However, a large Swedish study found similar rates of perioperative complications between those with well-compensated (asymptomatic) cirrhosis and without cirrhosis who underwent cholecystectomy. The main postoperative complication that contributed to the difference seen between the two groups was the increased need for blood transfusions, likely related to the increased bleeding tendency in cirrhotic patients. Therefore, current evidence suggests that laparoscopic cholecystectomy is a safe and effective treatment for symptomatic gallstone disease in patients with compensated and mildly decompensated cirrhosis (CTP class A and B) [55, 56].

### Umbilical hernia repair

Umbilical hernia occurs in 20% of patients with cirrhosis complicated with ascites and has the potential to enlarge rapidly due to increased intra-abdominal pressure [57]. Ascites can greatly complicate surgical repair, as it results in increased rates of wound infection, evisceration, and peritonitis. Therefore, management of ascites is key to reducing postoperative complications and hernia recurrence. Ideally, ascites is medically managed with loop diuretics and mineralocorticoid receptor antagonists, but sometimes, transjugular intrahepatic portosystemic shunt (TIPS) may be necessary to control ascites (though there are insufficient data to recommend TIPS to decrease operative risk). Early detection of the presence of an umbilical hernia is also important to allow time to manage ascites and plan for surgery. One retrospective cohort study of patients with cirrhosis who underwent umbilical hernia repair found that emergency surgery had higher postoperative mortality than elective surgery [58]. In patients who are likely to be transplanted in 3–6 months, hernia repair should be performed during the time of transplant [57]. Studies have also shown that hernia repair with mesh is associated with a lower recurrence rate but higher surgical site infection when compared with conventional fascial suture, and therefore the risks and benefits of each should be carefully considered by the surgical team [59].

### Bariatric surgery

The obesity epidemic has resulted in an increase in patients with CLD and cirrhosis secondary to MASLD. Furthermore, obesity in patients with cirrhosis is a major complication for hepatic decompensation, portal vein thrombus, hepatocellular carcinoma, and the development of acute or chronic liver failure. Evidence suggests that weight loss is beneficial in improving or reversing complications of CLD, but management of obesity is often challenging. Bariatric surgery is one of the most effective and durable methods for managing obesity and can be considered for patients with well-compensated cirrhosis. In fact, the AGA released best-practice guidelines on this topic [60]. They recommend considering bariatric surgery in well-compensated cirrhotic patients who are obese to reduce the risk of hepatocellular carcinoma. Prior to surgery, they recommend evaluating for clinically significant portal hypertension (CSPH), as this increases the postoperative mortality. Again, portal hypertension can be measured by using transjugular pressure measurements, with a hepatic vein portal gradient (HVPG) of <10 mmHg considered safe. If there is no CSPH, then bariatric surgery may be performed at institutions with expertise in cirrhosis. If, however, patients do have CSPH, bariatric surgery, including endoscopic bariatric therapy, is contraindicated. The type of bariatric surgery is also important to consider. The AGA states that laparoscopic sleeve gastrectomy is likely superior to Roux-en-Y gastric bypass because it preserves endoscopic access to the stomach and biliary tree, while also not inducing malabsorption. Furthermore, Roux-en-Y gastric bypass may complicate liver transplant [61]. More recently, evidence supports sustained weight loss and graft survival in obese patients who undergo bariatric surgery at the time of liver transplant [62]. Therefore, combined sleeve gastrectomy and liver transplant should be considered in this patient population.

### Complications

Several factors contribute to increased surgical risk in patients with cirrhosis, including malnutrition, acute kidney injury due to decreased effective intravascular volume, and coagulopathy potentially causing bleeding and thromboembolism. Patients with cirrhosis are prone to complications postoperatively as well, including difficult wound healing, multiorgan failure, infection, and decompensating events of cirrhosis such as ascites, hepatic encephalopathy, and portal hypertensive bleeding. Studies have examined whether the presence of TIPS reduces surgical risk in patients with cirrhosis. One retrospective cohort study found that the presence of a TIPS (placed 3 months before surgery) was associated with a lower incidence of in-house mortality and liver transplant after surgery [63]. This study also noted that, although the predicted mortality risk according to the VPS was higher for those with a TIPS than for those without, the incidence of in-house mortality and liver transplant was lower across all strata of risk prediction. Although current guidelines do not recommend TIPS prior to surgery to reduce risk, further investigation is warranted. Below we will describe several specific complications and their perioperative management.

#### Ascites

The presence of ascites is a contraindication to abdominal surgery because it results in increased rates of wound infection, evisceration, and peritonitis, and thus should be actively controlled prior to surgery if possible [57]. Strategies to achieve this include restriction of dietary sodium, strict fluid restriction, initiation of loop diuretics and mineralocorticoid receptor antagonists, and lastly TIPS or transplant [52]. If the control of not ascites possible, then elective abdominal surgery is contraindicated. For non-abdominal surgeries, uncontrolled ascites may impair lung function, increasing the risk of aspiration during anesthesia induction and delaying the recovery of pulmonary function postoperatively [13]. Symptomatic patients, including those with shortness of breath due to ascites, should receive a large-volume paracentesis preoperatively with an infusion of 6–8 g of albumin for each liter removed [64]. If performed the day before surgery, it may improve ventilation and volume control, as well as decrease infection risk, especially spontaneous bacterial peritonitis. In addition, thoracentesis should only be performed in patients with moderate to large pleural effusions, including hepatic hydrothorax, that cause impaired pulmonary function [15]. Postoperatively, excess intravenous fluid and blood products may cause volume overload and increased portal pressures. Therefore, intravenous fluid resuscitation and transfusions should be utilized to maintain a set measure, such as a hemoglobin of >7 g/dL [65].

#### Infections

Patients with cirrhosis with preoperative infections are susceptible to increased mortality. Clinicians should keep a high index of suspicion for spontaneous bacterial peritonitis (SBP) for any patient who clinically worsens and a diagnostic paracentesis should be obtained [64]. Empiric antibiotics should be given accordingly for any concern about infection and subsequently narrowed based on culture results. Patients with a history of SBP or low ascitic total protein may resume their SBP prophylaxis after surgery. In patients unable to tolerate oral intake postoperatively, third-generation cephalosporins may be given in the interim [13].

#### Coagulopathy

Due to variations in coagulation factors as well as anticoagulants, patients with cirrhosis are susceptible to both increased bleeding and thrombotic events [66]. Specifically, thrombocytopenia and decreased production of fibrinogen and coagulation factors increase the risk of bleeding in cirrhosis; however, decreased production of protein C and S increases the risk of thrombosis. Abnormal function of vascular endothelial cells increases von Willebrand factor and factor VIII [65]. Due to the increased risk of thrombosis, patients with cirrhosis should be placed on venous thromboembolism prophylaxis if possible. This could include unfractionated heparin, given its shorter duration of action, or sequential compression devices if chemical prophylaxis is contraindicated [67]. In patients with cirrhosis, the INR does not correlate with bleeding risk [65]. Therefore, there are no recommendations for protocol transfusions to target a particular INR in this population, as it has not been effective for decreasing perioperative bleeding [13]. Prophylactic preoperative fresh frozen plasma (FFP) based on INR alone is not recommended and its additional volume may cause worsening portal hypertension and increasing risk of further complications [65]. In the setting of significant malnutrition or biliary obstruction, vitamin K may be utilized [15].

Platelet levels of >50,000/mL are sufficient for clot formation in patients with cirrhosis [65]. While this is enough for most surgeries, platelets of >100,000/mL are suggested for neurosurgery or neuraxial anesthesia [13]. Similarly to FFP for INR, prophylactic transfusions for platelets above these levels may also cause volume overload or thrombosis [13]. In addition, thrombopoietin receptor agonists have been utilized in patients with thrombocytopenia in the setting of cirrhosis [65]. Eltrombopag has been used in chronic hepatitis C cirrhosis to increase platelet levels during interferon therapy [68]. Avatrombopag has been utilized to raise platelet levels prior to surgery, but bleeding events did not decrease while the risk of thrombosis increased [69].

Decreased fibrinogen levels of <100 mg/dL in patients with cirrhosis may inhibitor clot forming and increase the risk of bleeding [70]. While research is limited, cryoprecipitate may be utilized to raise fibrinogen levels prior to high-risk surgeries. Low-volume cryoprecipitate may replete fibrinogen while having a lower risk of volume overload [13].

Thromboelastography and rotational thromboelastometry have decreased the need for transfusions in patients with cirrhosis [71]. These viscoelastic tests should be considered before and during surgery to assist in indications for transfusions and prevent volume overload.

#### Hepatic encephalopathy

Hepatic encephalopathy is a clinical diagnosis of transient cognitive impairment in cirrhosis and is a manifestation of hepatic decompensation. Risk factors include electrolyte disorders, gastro-intestinal bleeding, constipation, analgesic medications, and infection such as SBP. Any patient with hepatic encephalopathy should be assessed for these risk factors and the underlying inciting factor should be managed accordingly. Hepatic encephalopathy is treated with oral or rectal lactulose titrated to about three bowel movements daily, as well as rifaximin as additional therapy. Trending ammonia levels is not recommended. Treatment response is clinically observed [72]. Patients with hepatic encephalopathy likely have CSPH, which increases surgical risk. As mentioned previously, some scoring systems such as CTP take the presence of hepatic encephalopathy into account and others such as MELD-Na do not.

#### Malnutrition

Patients with cirrhosis undergoing abdominal surgery are at increased risk of poor outcomes when they also have protein-calorie malnutrition or sarcopenia [15]. To diagnose malnutrition, patients must have at least one phenotypic criterion (weight loss, low body mass index, and reduced muscle mass) and one etiologic criterion (reduced food intake or assimilation, and inflammation or disease burden). Ongoing alcohol use increases catabolism as well, worsening malnutrition. Due to decreased gluconeogenesis in cirrhosis, fasting should be minimalized and glucose should be monitored perioperatively [73].

Patients with sarcopenia or decreased muscle activity may benefit from increasing nutrition prior to elective surgeries. While specific diets have not shown benefit, increased protein intake is recommended—about 1.2–1.5 g of protein/kg of body weight daily. Protein restriction does not decrease the risk of hepatic encephalopathy and should be avoided. If possible, enteral nutrition should be utilized over parenteral nutrition, with the help of nasogastric or nasojejunal tubes if needed. Patients with ascites or varices should refrain from percutaneous endoscopic gastric tube placement due to increased risk of complications [73].

#### Renal dysfunction

Patients with cirrhosis are at increased risk of mortality with renal dysfunction. Renal impairment is a sequelae of portal hypertension, expressed by the incorporation of creatinine as a factor of the MELD score. Close monitoring of kidney function postoperatively is recommended [15].

In the postoperative setting, common causes of renal dysfunction in patients with cirrhosis include acute tubular necrosis, intravascular volume depletion, and hepatorenal syndrome [64]. Evaluation for acute kidney injury includes complete blood count and serum electrolytes including magnesium, phosphorus, and calcium. In addition, urinalysis with microscopy, urine electrolytes, and retroperitoneal ultrasound should be obtained. Sufficient intravascular resuscitation is recommended in the postoperative setting to maintain adequate renal perfusion. This includes the utilization of albumin infusion after large-volume paracentesis. Nephrotoxic agents should be avoided as well. Hepatorenal syndrome—a diagnosis of exclusion—may be suspected if the albumin volume challenge fails to improve renal function. Prompt treatment of hepatorenal syndrome, which may include vasoactive medications, is critical to avoid worse outcomes [64].

#### Decompensation after surgery

While risk stratification prior to surgery helps to minimize postoperative complications, patients may decompensate to the degree that their best outcome is with liver transplant. Patients undergoing surgery with a MELD of ≥15 should undergo formal evaluation for liver transplant prior to surgery [13]. Elective surgeries in those at highest risk of a poor outcome should be delayed until liver transplant. If the patient is not a liver transplant candidate despite having a high MELD, then clinicians should discuss long-term prognosis and likely avoid elective surgery.

### Key takeaways

- Patients with cirrhosis are at high risk of perioperative complications, including ascites, volume overload, encephalopathy, bleeding, thromboembolism, renal impairment, decompensation, and death, after any invasive surgical procedure.
- All patients with cirrhosis should be risk-stratified prior to undergoing surgical procedures. Patients with Child–Pugh class C or MELD of >20 points have a high risk of decompensation and mortality.
- The type of surgery impacts perioperative risk, with abdominal wall and orthopedic surgeries being lower-risk than intra-abdominal and intrathoracic surgery. When able, laparoscopic surgery is preferred to an open approach.
- In patients who have high perioperative risk (MELD > 15), liver transplant evaluation should be completed prior to surgery.

### Non-cirrhotic liver disease

Studies have shown that patients with non-cirrhotic CLD overall have fewer perioperative risks than patients with cirrhosis [52]. Therefore, the etiology for each CLD should be considered when evaluating patients preoperatively [74].

### MASLD

There are a growing number of patients who have CLD but not cirrhosis. The largest of this cohort is patients with MASLD. MASLD increases a patient’s risk of diabetes as well as atherosclerotic cardiovascular disease. Therefore, patients may benefit from increased cardiac risk evaluation as well as monitoring for cardiac events perioperatively [75]. Recent studies have shown that glucagon-like peptide-1 (GLP-1) agonists can reduce liver fat content and help to resolve steatohepatitis [76]. However, GLP-1 agonists may impact surgical risk by delaying gastric emptying and controversy exists on how to best manage patients on these medications perioperatively. Bariatric surgery may also be considered in obese patients and was shown to lead to complete resolution of MASLD in obese patients in a meta-analysis of 32 cohort studies [77]. Patients with MASLD who do not have advanced fibrosis should be assessed for surgical risk independently of their liver disease.

### Chronic viral hepatitis

While non-cirrhotic chronic viral hepatitis is not associated with increased perioperative risk, continued adherence to antiviral therapy perioperatively is important to help lower viral load as well as avoid drug resistance [15]. In the event that therapy needs to be held for the surgery, elective surgery may be delayed until patients complete the hepatitis C treatment course [25].

### Autoimmune hepatitis

Autoimmune hepatitis is a condition in which the body’s immune system attacks liver hepatocytes and causes inflammation and subsequent fibrosis [78]. Serologic markers required for the diagnosis of autoimmune hepatitis include antinuclear antibodies, smooth muscle antibodies, and antibodies to liver-kidney microsome type 1 [78]. If left untreated, autoimmune hepatitis can lead to cirrhosis and liver failure. Like other autoimmune conditions, autoimmune hepatitis is treated with immunosuppressive medications. The American Association for the Study of Liver Diseases (AASLD) guidelines recommend monotherapy with prednisone or combination therapy with prednisone and azathioprine for induction, followed by steroid-sparing maintenance therapy if possible with azathioprine or mycophenolate mofetil [79]. These immunosuppressive medications must be continued long-term to avoid disease relapse but, in patients undergoing surgery, this may increase the risk of postoperative surgical site infection and delay wound healing [80]. Therefore, perioperative management of immunosuppression can be challenging and requires careful risk–benefit analysis. Currently, there are no guidelines regarding the management of immunosuppressive medications perioperatively in patients with autoimmune hepatitis. However, studies have evaluated surgical risk in rheumatologic patients on immunosuppressive medications. For azathioprine, the guidelines recommend either continuing it through the perioperative period or holding 1 day prior to surgery and resuming 3 days after surgery [81]. The guidelines recommend considering the type of surgery, co-morbidities, and severity of disease when deciding the appropriate strategy because pausing immunosuppression can result in a flare and negatively impact the surgical outcome [81]. In patients who are stable on an immunosuppressive regimen and undergoing lower-risk surgery such as total hip or knee arthroplasty with few other co-morbidities, it may be reasonable to continue immunosuppression throughout the perioperative period. In the case that a patient is recently diagnosed with autoimmune hepatitis, surgery should be deferred until biochemical remission (normal transaminases and IgG) is achieved. Postoperatively, some patients may require stress dose steroids, depending on the type of surgery [74].

### Primary biliary cholangitis and primary sclerosing cholangitis

Primary biliary cholangitis and primary sclerosing cholangitis (PSC) may predispose patients to hyperlipidemia; however, it is unclear whether they increase the risk of cardiac events in the absence of other risk factors [82]. Patients using naltrexone to manage pruritus should stop preoperatively and are at risk of increased sensitivity to opioids for analgesia [83]. PSC is also associated with an increased risk of gallbladder cancer. Therefore, the AGA recommends close surveillance for cholangiocarcinoma and gallbladder cancer every 6–12 months in its guidelines [84]. If a gallbladder mass is seen, even if <1 cm in size, the AASLD recommends cholecystectomy as treatment if the liver disease permits [85]. As the guidelines mention, the extent of underlying liver disease can be a limitation to surgery and thus early surveillance for gallbladder cancer prior to the development of PSC cirrhosis is important in having definitive treatment options and minimizing surgical risk.

### Genetic liver diseases

Hereditary hemochromatosis increases the risk of cardiomyopathy, diabetes, as well pituitary disease. Patients may benefit from echocardiography to assess for structural heart disease and consultation with an endocrinologist if there is any concern for Cushing’s disease or uncontrolled diabetes [86].

Wilson’s disease places patients at increased risk of delirium perioperatively and they may benefit from early preventative precautions. D-penicillamine can interfere with postoperative wound healing [87].

Alpha-1 antitrypsin deficiency increases the risk of chronic obstructive pulmonary disease [88]. Patients may benefit from pulmonary evaluation prior to surgery with chest X-ray, computed tomography scan, oxygen saturation, and pulmonary function testing [15].

### Key takeaway

- Patients with non-cirrhotic liver disease have lower perioperative risk than those with cirrhosis; however, the etiology of the liver disease must be considered to best optimize patients prior to surgery.

## Future directions

In patients with CSPH but low MELD-Na, preoperative TIPS has been considered to reduce perioperative risk. However, this has not yet been recommended by guidelines. Research has shown that the degree of portal hypertension can accurately predict postsurgical outcomes, with a HVPG of >16 mmHg considered high-risk [89]. Still, only few single-center reports have been published regarding prophylactic TIPS prior to elective surgery [90].

One study showed that those with cirrhosis who had the presence of a TIPS prior to surgery had lower rates of postoperative acute-on-chronic liver failure than those who did not have a TIPS, especially in those with a Chronic Liver Failure Consortium–Acute Decompensation (CLIF-C AD) score of >45 [91]. Of note, these patients had a TIPS placed for difficult-to-control ascites or bleeding, not specifically for the purpose of potentially reducing surgical risk. In the limited studies, the timing of the TIPS was variable, ranging from 1 week to 3 months prior to surgery, and there is currently no consensus as to when is the optimal timing for surgery after TIPS. Furthermore, the HVPG measurement may change from the time of TIPS placement to the time of surgery, and this needs to be further investigated.

Overall, TIPS can theoretically reduce surgical risk by decreasing the chances of major bleeding and decrease the risk of infection by reducing ascites. However, TIPS can also increase the chances of hepatic encephalopathy, as blood with toxins is shunted to the brain. Therefore, further investigation into prophylactic TIPS prior to elective surgery, appropriate patient selection, and the impact on surgical outcomes should be conducted to better guide perioperative risk stratification.

### Key takeaway

- Studies suggest that preoperative TIPS may reduce surgical risk but further studies must be conducted, as this is not currently recommended by guidelines.

## Limitations

There are many risks associated with operating on patients with CLD. While multiple scoring tools exist to help quantify individualized perioperative risk and societies have released guidelines to help clinicians minimize risk to patients, these systems and guidelines are limited by the fact that most studies are based on observational and retrospective data with heterogeneity of the severity of liver disease, type of surgery, hospital resources, and urgency of surgery.

## Conclusions

With the increasing prevalence of CLD in the USA and worldwide, it is more likely that patients with CLD are considered for surgery. Patients with acute liver diseases should wait until after the resolution of acute liver disease before proceeding with surgery. Various perioperative risk assessment tools exist; they take into account the lab values, etiology of the liver disease, and type of surgery to help quantify risk. Each scoring system has a different level of evidence and different strengths and weaknesses. A patient’s liver disease should be carefully reviewed and optimized prior to proceeding with surgery, as surgery poses a high risk of complications and hepatic decompensation.

### Key takeaways

- Prior to surgery, clinicians should take a detailed history and perform physical examinations that aim to identify the presence of acute liver disease, CLD, and/or portal hypertension.
- Multiple scoring tools exist to aid clinicians in assessing perioperative risk in patients with liver disease. Each scoring tool has its strengths and weaknesses, and they can be used in conjunction to better gauge an individual patient’s perioperative risk.
- Acute liver diseases including ALF, AH, and DILI are contraindications to surgery. In these instances, surgery should be deferred until resolution of these acute conditions.
- Patients with cirrhosis are at high risk of perioperative complications, including ascites, volume overload, encephalopathy, bleeding, thromboembolism, renal impairment, decompensation, and death, after any invasive surgical procedure.
- All patients with cirrhosis should be risk-stratified prior to undergoing surgical procedures. Patients with Child–Pugh class C or MELD of >20 points have a high risk of decompensation and mortality.
- The type of surgery impacts perioperative risk, with abdominal wall and orthopedic surgeries being lower-risk than intra-abdominal and intrathoracic surgery. When possible, laparoscopic surgery is preferred to the open approach.
- In patients who have high perioperative risk (MELD > 15), liver transplant evaluation should be completed prior to surgery.
- Patients with non-cirrhotic liver disease have lower perioperative risk than those with cirrhosis; however, the etiology of the liver disease must be considered to best optimize patients prior to surgery.
- Studies suggest that preoperative TIPS may reduce surgical risk but further studies must be conducted, as this is not currently recommended by guidelines.
